# The Chicago Gynecological Society, Meeting of September 18th

**Published:** 1885-10

**Authors:** 

**Affiliations:** 2330 Indiana avenue


TRANSACTIONS OF THE CHICAGO GYNECOLOGICAL SOCIETY.

*Regular meeting, Friday evening, September 18th, 1885.*

I. DUDLEY—*Remarks upon Abdominal and Gynecological Surgery in England, Scotland and Heidelberg.*

The PRESIDENT, DR. H. P. MERRIMAN, in the chair.

PROFESSOR E. C. DUDLEY made some informal remarks relative to his observations in gynecological and abdominal surgery, during a summer holiday, in Europe. His observations were confined to the work of a few operators in England, Scotland and Heidelberg.

In Heidelberg, he called upon PROFESSOR KEHRER. PROFESSOR DUDLEY inspected the hospital and saw evidence of considerable work in abdominal surgery. PROFESSOR KEHRER'S laboratory gave evidence of active research into gynecological bacteriology. His work bore the stamp of thoroughness and efficiency. PROFESSOR KEHRER is a medium-sized man, frail and delicate, with a large head and small body.

A call upon DR. BANTOCK, at his office, No. 18 Harley Street, W., London, resulted in a pleasant hour's conversation upon subjects pertaining to ovariotomy and hysterectomy. Patients at the Samaritan Hospital sometimes die within twenty-four hours after laparotomy, with a high temperature. This condition was called acute sepsis by certain systematic writers. DR. BANTOCK thought the true pathology of the condition was unknown, and was not satisfied with the term, acute sepsis. PROFESSOR DUDLEY saw DR. BANTOCK operate at the Samaritan Hospital. The first operation was the removal of a small, solid ovarian tumor. The remaining ovary and tube, although normal, were removed on account of a small intra-mural, uterine fibroid. The striking feature of the operation was great rapidity without haste. DR. BANTOCK caught up the edges of the peritoneum with small compression forceps, so that these edges were drawn up towards the cutaneous edges, and were held in this position by the weight of the instrument against the abdominal surface. This manœuvre greatly facilitated the passage of the sutures. The pedicle was secured by means of silk ligature, applied in the operator's peculiar figure-of-eight turns.

In closing the wound, a needle of ovoid shape, curved on the edge, instead of on the flat, was employed. This needle combines the maximum of strength with the minimum of size. Two or three sutures were passed through at each angle ot the wound. Their ends were joined by knots. An assistant, passing the index finger of each hand through the loops thus formed, made traction at each angle of the wound, in such a manner as to draw its sides into contact, and to lift the peritoneal edges nearer to the surface. The introduction of the remaining sutures was in this manner greatly facilitated. The sutures were so closely passed that no superficial stitches were required. They were made to include a very narrow margin of skin and peritoneum, and very little if any muscular tissue. Fine silk-worm gut was employed.

The ends of the sutures, on each side of the wound, were now grasped in lock forceps, which prevented them from being drawn out, or becoming tangled during the separation of the wound for the toilet of the peritoneum, which was most thorough, the entire cavity being rendered perfectly clean and dry. The lock forceps were then removed from the ends of the sutures, and the hands of the assistant substituted. The action was thus made on all the sutures, in the direction of the upper angle of the wound, and they were tied in order from below upward and cut short. This prevents tangling of the threads and otherwise facilitates tying. Antiseptics, throughout the operation, were conspicuous by their absence. The dressings were of the most simple character.

DR. BANTOCK kindly showed PROFESSOR DUDLEY over the hospital, which contained a number of convalescents from hysterectomy, ovariotomy and oöphorectomy. DR. BANTOCK'S exceptionally good results, in the last operation, are recognized throughout the world. His wonderful statistics in abdominal surgery are due to downright splendid operating. DR. MERIDITH, at the same time, was removing a tumor in another room, under the most extreme antiseptic conditions. The famous Samaritan Hospital is an unpretentious building, seemingly a large reconstructed dwelling, in the middle of a block, with houses joining on either side, and, like great men, has a modest appearance.

It is generally supposed in America that the Woman's Hospital in the State of New York, established by MARION SIMS in 1855, was the first of its kind in the world. This is a mistake. DR. SIMS himself, in a letter to DR. PROTHEROE SMITH, of London, dated July, 1883, accords to that gentleman the honor of having established the first hospital specially for the treatment of the diseases of women. This hospital, founded in 1842, is now a flourishing institution in London, and is called the Hospital for Women.

Its venerable founder visited Chicago a year ago. PROFESSOR DUDLEY again met him in London. His enthusiasm for the specialty, in which he has been a pioneer, continues, indeed, seems to increase with advancing years. He retains his official connection with the institution, as senior physician, and is still engaged in active practice. He was among the first, against bitter opposition, to advocate anæsthesia in labour. Efforts are now being made, with great promise ot success, to raise funds for the construction of a larger and more appropriate hospital building.

PROFESSOR DUDLEY visited Birmingham, in response to a polite telegraphic invitation from MR. LAWSON TAIT. On the train he occupied the same compartment with a sleek, well-fed, high-church London clergyman of the most conservative order, who intimated in no uncertain manner that the conservative people of London looked down upon the inhabitants of the radical city of Birmingham as a semi-barbarous community. So decided were his denunciations of the radical party in general, and of Birmingham in particular, which as the chief stronghold of radicalism always return JOHN BRIGHT and CHAMBERLAIN to Parliament, that PROFESSOR DUDLEY in an apologetic manner explained that he was only going into the jaws of the Philistine to witness an operation by a distinguished surgeon, from whom he hoped to learn something. The clergyman inquired who the surgeon was, and upon hearing the name of LAWSON TAIT, exclaimed : "O, I know all about him, he is just as bad as any of them ;" which means that MR. TAIT is a radical in politics, as he is in surgery.

MR. TAIT'S ridicule of antiseptics is well-known. His rapid method of operating conveys to the casual observer the idea of haste and almost of carelessness.

But closer observation very soon shows him to be one of those rare operators, where dexterity amounts almost to a sleight of hand. An ovariotomy, in his hands, does not impress the observer as a capital operation. It seems almost as trivial as opening an abscess. His methods of operating did not materially differ from those of DR. BANTOCK. In closing the wound he used but one needle, threaded with a piece of long silk, introducing this as if for a continuous suture, but did not draw the thread tight. After the introduction of the needle, he left a long loop before the reintroduction. Then, after taking the last stitch, he lifted the free loops of silk on the index finger, and severed them with the scissors, thereby converting the continuous into an interrupted suture. These were tied in the ordinary way, and the wound was dressed in a manner, which would be eminently acceptable to his most bitter antiseptic enemy.

During the day, MR. TAIT performed ovariotomy, lumbocolotomy, perineorrhaphy, and excised a urethro-vulvar cyst, besides attending to a large number of consultations, in one of which PROFESSOR DUDLEY accompanied him to a distance of forty miles. This was for him only a moderate day's work. It is indeed evident that no other man in England controls a larger practice in abdominal surgery.

MR. TAIT impressed PROFESSOR DUDLEY as a sincere man of exceptionally strong and positive character and very much in earnest. Like VIRCHOW he is politically inclined; indeed, his temperament is such that he cannot see things go on without having a hand in them. He has taken active part in the city government of Birmingham, and as PROFESSOR DUDLEY was informed, had already declined to stand for Parliament.

During a brief visit in Edinburg, PROFESSOR DUDLEY was pleasantly entertained by DR. THOMAS KEITH, who had just re-returned from a consultation with DR. HOMANS in Boston, but unfortunately DR. KEITH did not operate during this time, although a large number of patients were waiting for him at the Royal Infirmary. His son, DR. SKENE KEITH, kindly invited PROFESSOR DUDLEY to an ovariotomy, his forty-eighth operation. Up to this time, he had only lost one or two patients. His operation presented some interesting peculiarities. He used probe-pointed scissors of a peculiar pattern, instead of the director, in going down through the deeper layers of the abdominal walls. By pressing firmly against the adhesions with a sponge, at the point of their attachment to the cyst, he literally sponged them away from the tumor. It was surprising to note the facility with which rather firm adhesions were thus broken. It is much easier to tear them from the tumor with the sponge than to tear the tumor from the adhesions. The breaking of the adhesions in this way is also much more gentle, and in the opinion of DR. KEITH, diminishes the danger of shock.

The adhesions were ligatured with fine cat-gut as fast as they were divided. In passing the ligatures a forceps, similar to the ordinary compression forceps, was used. This instrument had blades more than an inch long, of very small diameter, terminating in sharp points, so sharp that when the blades were closed they could be thrust through any soft tissue like a large needle. Grasping the ligature in the point of these blades, the tissue to be ligatured was transfixed. The ligature was then pulled through and the forceps withdrawn.

The pedicle was transfixed and ligatured, with fine silk, in the same way.

The cautery, to which much of the elder KEITH'S success has been attributed, was not employed in this case, because the pedicle was very slender. The reason why the cautery, in the hands of other operators, has not proved a more perfect protection against hæmorrhage, becomes apparent to any one who has witnessed its application in the hands of DR. KEITE. The whole secret of his method is, first, in the powerful compression of the pedicle between the broad blades of a heavy Baker-Brown clamp; second, in the prolonged application of the red-hot cautery iron, not only to the pedicle, but, after this has been burned to the level of the clamp, also to the clamp itself. In this way the clamp becames so hot that the included portion of the pedicle is slowly and thoroughly cooked, so that when the instrument is removed, the end of the pedicle is thin and translucent, resembling a horny substance. Such a pedicle, in the experience of DR. KEITH, never gives trouble from oozing.

The wound was closed with fine silk sutures which had been boiled. Ten or fifteen pieces of silk were threaded at each end with very finely well tempered needles nearly three inches long, which were introduced on either side from within outward. Very small margins of peritoneum and skin were included in the sutures. DR. KEITH thought it a very common fault among operators to draw the stitches too tight in tying. The long fine needle used in closing the wound is superior. It makes a very small puncture, which never bleeds, and is so fine that it is easily pushed through by means of finger and thumb without needle forceps.

In the *American Journal of Obstetrics*, April, 1880, MARION SIMS had given a remarkable description of the Keith operation, which has exerted a powerful and beneficent influence upon the operation in America. PROFESSOR DUDLEY could add little except the gentle handling of the adhesions with the sponge, the ligature forceps and the peculiar long straight needles already mentioned.

The wonderful success without antiseptics recorded by the great Scotch ovariotomist, by DR. BANTOCK and by MR. TAIT, who have reduced the mortality almost to zero, must have great influence in fixing the value of Listerism so far as it relates to abdominal surgery. At any rate, incompetent operators can no longer venture with impunity upon these capital operations under the dangerous impression, that in some mysterious way, antiseptics will deprive a crude surgical performance of its greatest perils. Evidently it was not so much a question of Listerism as of removing the tumor with the least possible amount of operating, and in the shortest time consistent with careful attention to detail, and in the most gentle manner.

PROFESSOR DUDLEY, however, raised the pertinent question whether Listerism should be placed on trial before a court of abdominal surgeons, and whether, if found unnecessary in peritoneal surgery, it would be fair to condemn it in general. He thought that such a verdict could not be sustained by the facts, but that the antiseptic principle in surgery was destined to stand. Even the most violent opponents of antiseptics agreed that perfect cleanliness was essential. He knew of no other method by which cleanliness could be rendered so nearly absolute. Nor did the seeming ability of two or three of the most dexterous operators to do witout antiseptics prove that it might not be a useful aid to others. Clearly, the man who removes a tumor with the least operating and handling of the parts will require fewer preventive measures against inflammation and sepsis. Antiseptics, therefore, might be most valuable for an inexperienced operator, and, to say the least, an additional safeguard for any one.

Some American operators were now having about as good results as could be shown in Great Britian, which seemed to indicate that our former high mortality in this American operation had been due in reality to bad operating, and not, as many supposed, to climatic causes.

The minor gynecology of Great Britian had apparently made but little progress since the days of BENNETT and SIMPSON. The general impression prevails that on this side of the Atlantic we are going wild in the minor gynecological surgery. In response, we may now congratulate our English brethren that many of their leading gynecologists are already commencing to comprehend, to appreciate and to perform the American operations of Perineorrhaphy, Elytrorrhaphy and Trachelorrhaphy, and at the same time to lay aside in a measure the old *porte caustique*.

DISCUSSION.

DR. H. P. NEWMAN said that there were other reasons for the brilliant success of foreign laparotomists than those referred to by Dr. Dudley. Aside from the facility and expeditious manner of operating, acquired by large experience, a prime factor is the justifiable self-confidence of the operator and a responsive confidence inspired in the patient.

PROFESSOR W. W. JAGGARD thought that minor gynecological operations, as PROFESSOR DUDLEY termed them, were less frequent in the United Kingdom and the Continent than in America. PROFESSOR DUDLEY had made this general assertion, and he agreed with him. He did not, however, think the operative skill of British or Continental surgeons inferior to that of their American *confrères*. The indications for operative procedure do not exist in the United Kingdom and the Continent as in America. Laceration of the cervix and perineum are of much less frequent occurrence. The *cervix uteri* is usually effaced, and the external os is fully dilated before the application of the forceps. Manual dilatation is less frequently practiced. The bag of waters is not prematurely ruptured. Greater care is taken with the preservation of the perineum. In a word, obstetricians are better operators, and do not require so-called gynecological assistants.

DR. E. J. DOERING said that, in 1874, he had been present at ovariotomy and other operations, performed at the Samaritan Hospital by SIR SPENCER WELLS. He was particularly impressed with the extreme care exercised in admitting spectators to the operations, each visitor being required to sign a statement that he had not made an autopsy or attended a case of contagious disease for the two or three days preceding. He desired to know whether these regulations were still in force, and also if MR. LAWSON TAIT and DR. KEITH required similar restrictions.

The PRESIDENT asked the following questions:

1. "Was any treatment given to the patients to prepare them for the operation by any of the eminent gentlemen mentioned?"

2. "How were the patients covered during the operation, or was the whole abdomen left bare?"

3. "How was the evacuation of the cyst managed?"

4. "Was the patient turned upon her side to accomplish this, as DR. THOMAS sometimes does?"

The PRESIDENT suggested that all who desired should ask questions for further light before the general discussion began.

PROFESSOR CHRISTIAN FENGER replied to the question, raised by PROFESSOR DUDLEY, that antiseptic precautions might be more important in surgery, in general, than in abdominal surgery, where it looked as though more perfect methods of operating without antisepsis gave as good results as with antisepsis, as follows:

He thought that the abdominal, or rather peritoneal cavity, in respect to the antiseptic precautions, occupies a peculiar position in surgery. The danger from absorption of the poisonous antiseptics is far greater in the abdomen than in wounds. The ability of the peritoneum to absorb serous fluid and blood before it decomposes, to encapsulate foreign substances not capable of absorption—*ex. gr.*, rubber ligature —is perhaps somewhat greater than the ability of a wound in that direction, although it may be that there is some prejudice about this, as we have not as yet used silk ligatures extensively in general surgery.

As to the question, whether more perfect methods of operating without antisepsis would improve the results, or rather prevent inflammation and sepsis, he could say that outside of the peritoneum this question must as yet be answered in the negative.

In 1873, VOLKMAN, of Halle, introduced the Lister method of dressing and operating in his surgical clinics. In his report of the work done in 1873 (*Beiträge zur Chirurgie*, 1875), the antiseptic surgery had reduced inflammatory and septic complications following excisions, amputations, fresh penetrating articular wounds, fresh open fractures, to a minimum never before dreamt of, and all this in one year. In the broad field of surgery it is not possible that VOLKMANN or anybody else could improve the *technique* of operating, to the extent of having the results change all of a sudden in that way. No surgeon would dare, to-day, to excise, for example, a knee-joint, without antiseptic precautions in all the minute details, even if he employed all the latest improvements in the method of operating. Abdominal surgery is the only branch of surgery in which, as yet, the heavy operating has been done without antiseptic precautions.

W. W. JAGGARD, M. D., *Editor.*
2330 Indiana avenue,

